# Severe Post-partum Hypothyroidism Triggering Psychogenic Non-epileptiform Seizures, Myopathy, and Myxedema Coma

**DOI:** 10.7759/cureus.61318

**Published:** 2024-05-29

**Authors:** Brandon L Welborn, Benjamin B Zwain

**Affiliations:** 1 Neurology, Edward Via College of Osteopathic Medicine, Spartanburg, USA; 2 Neurology, Bon Secours St. Francis Health System, Greenville, USA

**Keywords:** functional neurological disorder, hypothyroid myopathy, post-partum thyroiditis, hypothyroid myxedema coma, psychogenic non-epilipetic seizures

## Abstract

This study details the development of severe post-partum hypothyroidism exacerbating psychogenic non-epileptiform seizures (PNES) and culminating in myxedema coma. A 29-year-old female with a history of anxiety, attention-deficit/hyperactivity disorder (ADHD), and post-partum depression presented with confusion, aphasia, and severe bilateral leg cramping five months following vaginal delivery. Initial laboratory tests indicated elevated creatine kinase (CK) levels, suggestive of non-traumatic rhabdomyolysis. Subsequent seizure-like episodes and the absence of epileptiform activity on the electroencephalogram (EEG) raised suspicions of PNES. Further investigation upon readmittance to the hospital revealed a thyroid-stimulating hormone (TSH) level of 216 mIU/L (range: 0.4-4.0 mIU/L), free thyroxine (T4) level of 0.2 ng/dL (range: 0.8-1.8 ng/dL), and a CK level of 2083 U/L (range in females: 30-150 U/L), indicating severe hypothyroidism with myopathy. Reintroducing levothyroxine (Synthroid), which was previously discontinued during pregnancy, rapidly resolved her symptoms, supporting suspicions that her non-epileptic seizures and myopathy were both caused by her underlying severe post-partum hypothyroidism. She was maintained on levothyroxine with only one seizure-like episode following hospital discharge. This case illustrates the importance of a thorough endocrine assessment in patients with neuropsychiatric presentations, particularly in the peripartum period. It highlights the potential for severe thyroid dysfunction to manifest as PNES, emphasizing the complexity of diagnosing and managing such cases. The findings advocate for a multidisciplinary approach to evaluating post-partum females with neurological and psychiatric symptoms and provide evidence for the link between thyroid disorders and PNES, advocating for a nuanced approach in similar clinical scenarios.

## Introduction

The interactions between endocrine disorders and neuropsychiatric conditions create a complex diagnostic challenge, especially in the context of psychogenic non-epileptiform seizures (PNES). This study focuses on the association between PNES and post-partum thyroid disease. PNES, a subset of functional neurological disorder (FND) characterized by its neurological presentation akin to epileptic seizures but without the corresponding electroencephalographic abnormalities, has been increasingly recognized as a neurological consequence influenced by both psychological and physiological factors [1]. The condition is more prevalent in females, with a notable link between PNES and thyroid dysfunctions, including both hypothyroidism and hyperthyroidism [2,3].

Thyroid disorders, particularly in the post-partum period, pose significant clinical concerns due to their impact on the neuropsychiatric health of patients. Thyroid disorders are remarkably prevalent in the post-partum period, with studies indicating that post-partum thyroiditis affects approximately 8% of females, often leading to transient or permanent hypothyroidism [4]. Furthermore, PNES, representing a significant portion of non-epileptic seizures, accounts for approximately 20-30% of cases seen in epilepsy monitoring units, emphasizing its clinical relevance in neuropsychiatric care [1]. The hormonal changes that occur after childbirth can also lead to various thyroid problems, such as the development of Hashimoto’s thyroiditis [4,5]. The pathophysiology behind thyroid-induced PNES is multifaceted, involving a disruption in oxidative stress balance and alterations in the gamma-aminobutyric acid (GABA)ergic system, which can exacerbate neuropsychiatric conditions [6,7]. This is further complicated by the cognitive and mood disturbances often associated with hypothyroidism, which can mimic or trigger psychiatric conditions [8-10].

This study describes a 29-year-old female who presented with myopathy and multiple seizure-like episodes and was diagnosed with PNES in the setting of severe post-partum hypothyroidism culminating in myxedema coma. This illustrates the critical role of a thorough endocrine evaluation in patients presenting with neuropsychiatric symptoms, particularly in the peripartum period. The rapid resolution of symptoms following the identification and treatment of the underlying thyroid disorder underscores the importance of a thorough and multidisciplinary approach in similar clinical scenarios. This study contributes to the growing body of evidence supporting the need for an integrated approach in the management of PNES, particularly in the context of thyroid dysfunction, highlighting the complex connection between neuroendocrine and psychosomatic factors in such presentations [11,12].

## Case presentation

A 29-year-old breastfeeding female, with a medical history of anxiety, attention-deficit/hyperactivity disorder (ADHD), childhood abuse, syncopal episodes with preceding rotational dizziness, and post-partum depression following parturition five months prior, presented to the emergency department (ED). The patient, currently taking escitalopram (Lexapro) 20 mg daily, was found to exhibit confusion, word-finding difficulty, and anxiety on initial examination, and self-reported intense bilateral leg tightness and cramping in her calves, quadriceps, and iliopsoas muscles. The cramps rated 10/10 in severity, had persisted for approximately 10 days, with a noted escalation over the past three days. She reported the cramps felt similar to the ones she had as a child playing sports. The patient stated her legs felt unusually heavy without any discernible loss of strength or movement. She reported that she felt “mentally distracted” earlier that day, asking her spouse the same questions multiple times. She denied any recent trauma, injury, or illicit drug use. Physical examination revealed bilateral 4/5 hip flexor weakness; however, ambulation was intact. Laboratory investigations revealed an elevated creatine kinase (CK) level of 778 U/L (range in females: 30-145 U/L). A complete blood count (CBC), comprehensive metabolic panel (CMP), and other pertinent labs yielded unremarkable results (Table 1). She was diagnosed with non-traumatic rhabdomyolysis; however, no underlying cause was identified at this time, and she was discharged home for outpatient follow-up.

**Table 1:** Pertinent laboratory values on initial presentation to the hospital. BUN: blood urea nitrogen; eGFR: estimated glomerular filtration rate; WBC: white blood cells; ALT: alanine aminotransferase; AST: aspartate aminotransferase

Component	Initial presentation	Reference range
Sodium	139 mmol/L	133-143 mmol/L
Potassium	3.7 mmol/L	3.5-5.1 mmol/L
Magnesium	2.0 mmol/L	1.8-2.4 mmol/L
Calcium	8.8 mg/dL	8.3-10.4 mg/dL
Glucose	83 mg/dL	65-100 mg/dL
BUN	13 mg/dL	6-23 mg/dL
Creatinine	0.78 mg/dL	0.6-1.0 mg/dL
eGFR	>90 mL/min/1.73 m^2^	>60 mL/min/1.73 m^2^
WBC	4.8 k/µL	4.3-11.1 k/µL
Total bilirubin	0.4 mg/dL	0.2-1.1 mg/dL
ALT	36 U/L	12-65 U/L
AST	56 U/L	15-37 U/L
Alkaline phosphatase	85 U/L	50-136 U/L
Albumin	3.8 g/dL	3.5-5.0 g/dL
Total protein	7.3 g/dL	6.3-8.2 g/dL

The following day, she returned to the ED via ambulance after experiencing syncope and witnessed 15 episodes of seizure-like activity characterized by shaking and jerking movements, each lasting between 1 and 8 min. She had mild post-ictal confusion and was not actively seizing when the ambulance arrived but subsequently experienced three seizure-like episodes on the way to the hospital. The patient's medical history was devoid of any personal or familial seizure disorders; however, three years prior, she experienced a convulsive-syncopal-like episode with reported post-ictal confusion following a head injury at work. Upon reevaluation in the ED, the patient was lethargic but responsive, with a CK level of 962 U/L (range in females: 30-145 U/L) thought to be due to her seizure-like movements. During her ED stay, she experienced three seizure-like episodes without urinary incontinence or tongue biting. Treatment included 2 mg/mL lorazepam (Ativan), 2 g intravenous (IV) magnesium, and a loading dose of 3 g of levetiracetam (Keppra), followed by a maintenance dose of 500 mg twice daily and she was admitted for continuous EEG monitoring.

Neurological examination revealed a slow response in answering questions, 4/5 strength in the deltoid muscles bilaterally, and 4+/5 strength in the remaining upper extremity muscles. Strength in the iliopsoas and quadriceps muscles was 4/5 bilaterally. Her cranial nerve examination, fundoscopic examination, muscle tone, and bulk were normal bilaterally, with no pathological reflexes or skin abnormalities noted. The patient exhibited normal mood and affect. A computed tomography (CT) head without contrast revealed no acute intracranial abnormalities or changes compared to a scan four years prior. A magnetic resonance imaging (MRI) head was performed and was unremarkable for any acute or chronic findings. Additional investigations, including chest radiography and laboratory tests for prolactin, magnesium, CBC, and CMP, were unremarkable (Table 2).

**Table 2:** Pertinent laboratory values on second presentation to the hospital. BUN: blood urea nitrogen; eGFR: estimated glomerular filtration rate; WBC: white blood cells; ALT: alanine aminotransferase; AST: aspartate aminotransferase

Component	Second presentation	Reference range
Sodium	141 mmol/L	133-143 mmol/L
Potassium	3.6 mmol/L	3.5-5.1 mmol/L
Magnesium	2.3 mmol/L	1.8-2.4 mmol/L
Calcium	8.9 mg/dL	8.3-10.4 mg/dL
Glucose	77 mg/dL	65-100 mg/dL
BUN	15 mg/dL	6-23 mg/dL
Creatinine	1.00 mg/dL	0.6-1.0 mg/dL
eGFR	>60 mL/min/1.73 m^2^	>60 mL/min/1.73 m^2^
WBC	6.1 k/µL	4.3-11.1 k/µL
Total bilirubin	0.5 mg/dL	0.2-1.1 mg/dL
ALT	1 U/L	12-65 U/L
AST	62 U/L	15-37 U/L
Alkaline phosphatase	91 U/L	50-136 U/L
Albumin	4.1 g/dL	3.5-5.0 g/dL
Total protein	8.0 g/dL	6.3-8.2 g/dL

The following morning, the patient experienced several seizure-like episodes characterized by thrashing, grunting, tensing, and shaking, immediately following complaints of increased muscle soreness. These episodes occurred without urinary incontinence or tongue biting, and she responded to questions immediately afterward. Continuous video-EEG monitoring during these events showed no epileptiform activity. By evening, her episodes intensified, involving tongue biting, back arching, and irregular flopping movements of her legs and trunk, each lasting over 5 min followed by post-ictal confusion and drowsiness. Her symptoms were alleviated with 2 mg of IV lorazepam. That night, she experienced multiple tonic-clonic-like episodes with tongue biting and was again treated with 2 mg IV lorazepam. Post-event, she was somnolent, but continuous EEG monitoring revealed no epileptiform activity.

Neurology suspected PNES based on her physiologically typical EEG on continuous monitoring and her psychological history. Levetiracetam was discontinued and the patient continued to experience similar events, with the longest event lasting 8 min, occurring while fully awake. Prolactin levels drawn immediately following one of these events were normal, supporting the diagnosis of PNES (Table 3). At discharge, the patient's CK levels had decreased to 311 U/L (range in females: 30-145 U/L).

**Table 3:** Comprehensive myositis laboratory profile including ENA JO-1 AB, aldolase, and ANA, with normal prolactin levels drawn immediately post-seizure. ANA: antinuclear antibody; ENA: extractable nuclear antigens

Component	Result	Reference range
ANA	Negative	Negative
ENA JO-1 Ab (AI)	<0.2	<1.0
Aldolase (U/L)	14.2	3.3-10.3
Prolactin (ng/mL)	17.8	<19.7

Two days later, on neurology follow-up, her myositis labs showed elevated aldolase levels (3.3-10.3 U/L) with negative results for anti-Jo-1 antibody (ENA JO-1 Ab) (0.0-0.9 AI) and antinuclear antibody (ANA) (<19.7 ng/mL) (Table 3). She described persistent muscle soreness, more pronounced on the left side, and difficulty with ambulation and rising from a seated position, but no lower leg pain. No seizure-like events or falls occurred since hospital discharge and cognitive behavioral therapy (CBT) was recommended for managing her PNES.

Three days post-discharge, the patient attended transitional care, reporting no new events since hospital discharge. That evening, her muscle pain intensified from 2/10 to 10/10, followed by four witnessed seizure-like episodes at home with post-ictal confusion. She was brought back to the ED via ambulance and despite receiving 5 mg of midazolam, the seizure-like activity persisted, and she was intubated during transport. Upon arrival, the patient was admitted to the intensive care unit (ICU) where initial investigations, including a urine drug screen (UDS), infectious workup, CBC, and CMP, were normal (Table 4). Repeat CT imaging, electrocardiogram (ECG), and electrolyte analysis were unremarkable. However, labs showed an elevated CK level of 2083 U/L (range in females: 30-145 U/L).

**Table 4:** Pertinent laboratory values on ICU admission. BUN: blood urea nitrogen; eGFR: estimated glomerular filtration rate; WBC: white blood cells; ALT: alanine aminotransferase; AST: aspartate aminotransferase

Component	ICU presentation	Reference range
Sodium	137 mmol/L	133-143 mmol/L
Potassium	4.5 mmol/L	3.5-5.1 mmol/L
Magnesium	2.0 mmol/L	1.8-2.4 mmol/L
Calcium	8.2 mg/dL	8.3-10.4 mg/dL
Glucose	69 mg/dL	65-100 mg/dL
BUN	10 mg/dL	6-23 mg/dL
Creatinine	1.00 mg/dL	0.6-1.0 mg/dL
eGFR	>60 mL/min/1.73 m^2^	>60 mL/min/1.73 m^2^
WBC	7.0 k/µL	4.3-11.1 k/µL
Total bilirubin	0.4 mg/dL	0.2-1.1 mg/dL
ALT	44 U/L	12-65 U/L
AST	39 U/L	15-37 U/L
Alkaline phosphatase	85 U/L	50-136 U/L
Albumin	3.5 g/dL	3.5-5.0 g/dL
Total protein	6.9 g/dL	6.3-8.2 g/dL

The next day, she was extubated following stabilization and initiated bilevel positive airway pressure (BiPAP) therapy. During an interview, she developed a scanning gaze, became unresponsive, and opened her eyes only briefly with marked stimulation. Her blood glucose levels were noted to be severely low at 52 mg/dL (65-100 mg/dL), and she was administered an infusion of dextrose 10% (D10). The patient continued to be somnolent, with a scanning gaze, waxing and waning diffuse tremors, and bilateral brisk reflexes in her lower extremities, accompanied by a bilateral 2-beat ankle clonus.

Further inquiry with the patient’s spouse revealed that the patient experienced hair loss, weight gain, fatigue, lower extremity weakness and pain, and constipation following her recent vaginal delivery. During her pregnancy, she stopped taking levothyroxine, which was prescribed for new-onset hypothyroidism, due to the development of a rash. However, the patient’s spouse reported her TSH levels returned to normal post-pregnancy. In the ICU, her TSH level was 216 mIU/L (range: 0.4-4.0 mIU/L), with a T4 level of 0.2 ng/dL (range: 0.8-1.8 ng/dL), which went unchecked during her recent hospitalization. A complete list of her thyroid labs, including TSH, T4, and triiodothyronine (T3), are listed in Table 5. Levothyroxine was then initiated at a bolus dose of 200 µg and reduced to 100 µg daily for long-term treatment. No seizure-like episodes occurred following the initiation of levothyroxine and normalization of her TSH level.

**Table 5:** Timeline of thyroid function test results including TSH, free T4, T3, and TPO levels from before admission, during admission, and subsequent follow-up appointments. TSH: thyroid-stimulating hormone; TPO: thyroid peroxidase

Component	May 07, 2019	May 06, 2020	September 19, 2023	September 20, 2023	October 03, 2023	November 17, 2023	November 29, 2023	May 02, 2024	Reference range
TSH (mIU/L)	3.980	2.417	216.00	N/A	13.5	2.91	3.190	1.65	0.4-4.0
Free T4 (ng/dL)	1.09	N/A	0.2	N/A	1.0	N/A	1.0	1.7	0.8-1.8
T3 (nmol/L)	N/A	N/A	N/A	0.8	N/A	N/A	N/A	N/A	0.9-2.8
TPO (IU/mL)	N/A	N/A	N/A	N/A	>600	N/A	N/A	N/A	<35

Within 24-h, she made a complete recovery back to baseline. Over three days in the ICU, her CK levels trended downward (Table 6). The patient's clinical presentation, characterized by hypothyroid myopathy, aligned with her medical history, reported symptoms, and laboratory findings, strongly indicated a progression to myxedema coma. After consulting with endocrinology, it was concluded that her symptoms indicated severe hypothyroidism which led to myxedema coma, and neurology determined that this was the underlying trigger for her non-epileptic seizures and myopathy.

**Table 6:** CK levels during initial hospitalization, outpatient follow-up, and ICU stay in a female with hypothyroidism and seizure-like episodes. CK: creatine kinase

Component	Initial hospitalization				Outpatient	ICU admission				Reference range (female)
Date	September 13, 2023	September 14, 2023	September 15, 2023	September 16, 2023	September 18, 2023	September 19, 2023	September 20, 2023	September 21, 2023	September 22, 2023	
CK (U/L)	962	798	472	311	236	2083	2487	1503	949	30-145

Three days post-ICU admission, the patient was discharged on 100 µg of levothyroxine daily while continuing her home escitalopram dose. At her primary care visit four days post-discharge, the myopathic pain had mostly resolved, rated at 2/10, and there were no further reports of seizure-like activity. Upon endocrinology, follow-up one week post-discharge, mild thyromegaly was noted along with positive thyroid peroxidase (TPO) at >600 IU/mL (<35 IU/mL), suggesting an autoimmune component to her symptoms (Table 5). A subsequent ultrasound of her thyroid glands found no evidence of hypervascularity or suspicious nodules. She was diagnosed with severe post-partum hypothyroidism, resulting in myxedema coma, myopathy, and complicated by PNES exacerbations. The elevation in CK on initial presentation and the ICU admission was most likely due to worsening seizure-like occurring outside of the hospital.

Two months post-discharge, at endocrinology follow-up for a thyroid laboratory assessment, she stated she was feeling “different and off” with generalized malaise over the previous several days. She had not experienced any seizure-like events since discharge from the ICU. During the office visit, she expressed anxiety regarding the blood draw procedure and subsequently experienced four tonic seizure-like episodes, which resolved prior to arriving at the ED. Following this event, she did not experience any more seizure-like episodes. Notably, the patient's myopathy resolved, and her thyroid laboratory results remained stable on follow-up appointments (Table 5).

## Discussion

This case of a 29-year-old female experiencing PNES and myopathy secondary to severe post-partum hypothyroidism provides further insight into the interaction between neuroendocrine and psychosomatic factors. PNES, also referred to as functional seizures or dissociative seizures, presents as paroxysmal behavioral, motor, or sensory changes akin to epileptic seizures but lacking the corresponding electroencephalographic abnormalities typical of epilepsy [1]. PNES is a subset of FND characterized by pathophysiological abnormalities in neural circuits, leading to impairments in cognitive domains like agency, emotional processing, and attention. Self-agency, the belief that one initiates and controls movements or thoughts, is compromised in FND patients, resulting in discordance between one’s will and actions [13]. In this case, the patient's muscle pain could have aggravated a preexisting FND. The emergence of PNES in this patient, particularly following childbirth, likely reflects the impact of her hypothyroid state on neural circuitry, consistent with the cognitive disruptions typical in FND cases.

PNES, often influenced by psychological factors and a history of abuse, has a prevalence ranging from 2 to 33 per 100,000 persons [14]. Diagnostic challenges in PNES arise due to its occasional coexistence with epileptic seizures, which in one study was observed in 5.2% of cases in epilepsy monitoring units [15]. Additionally, PNES accounts for about 11% of convulsive seizure cases in emergency departments, and comorbid epilepsy is present in 14% of PNES patients, as shown in two population-based studies [16,17]. Functional seizures are more common in females, with a 3:1 ratio to males, and are strongly linked to thyroid problems such as hypothyroidism and hyperthyroidism; however, they necessitate a thorough diagnostic approach, including video EEG, to differentiate functional seizures from epilepsy [2,3]. The differentiation is complicated as PNES can mimic other medical conditions, displaying features like asynchronous movements, closed eyes, crying, stuttering, side-to-side head movements, and pelvic thrusting, which are atypical of epileptic seizures [18]. PNES often occurs after acute stressors and is linked to numerous comorbid disorders typically associated with a history of physical or sexual abuse, which presents a unique challenge in treatment [1,3]. In one meta-analysis, cognitive-behavioral therapy showed an 82% reduction in seizure frequency, but the efficacy of antidepressants in this disorder has not been proven [19].

Overt hypothyroidism often manifests in patients as slowed thought and speech, reduced attentiveness, and apathy, which can lead to a misdiagnosis of depression. It typically affects cognitive functions such as intelligence, attention, memory, language, executive function, and psychomotor function, and in extreme cases, agitation and psychosis have occurred [8]. Hypothyroid patients often exhibit higher anxiety or depression scores, which levothyroxine therapy sometimes partially alleviates, and some cognitive symptoms may persist despite this treatment [9,10]. Hypothyroidism is also associated with an increased prevalence of psychiatric diagnoses and changes in brain structure, as evidenced by imaging studies showing reduced hippocampal volume and cerebral blood flow [20]. Therefore, serum TSH testing is crucial in patients with overlapping affective and cognitive symptoms since mood and cognitive changes in overt hypothyroidism have the potential to trigger or worsen PNES in predisposed individuals.

The significant association between PNES and thyroid disorders, as evidenced by a recent study showing a higher prevalence of thyroid disorders in PNES patients compared to those with epilepsy, underscores a potential neurobiological link [7]. This connection is supported by the role of thyroid hormones in brain development and function, including neuronal maturation and gene expression modulation. Thyroid hormone receptor mutations are associated with various cognitive and emotional dysfunctions. Notably, these hormones impact limbic regions, which are crucial for emotional processing and are often implicated in the development of PNES. Therefore, hypothyroidism commonly linked with emotional disorders may exacerbate pathological responses to trauma, and due to the altered resting-state brain network connectivity present in patients with PNES, this reinforces the connection between thyroid disorders and PNES [7].

Severe hypothyroidism can contribute to the onset of seizures through various mechanisms within the brain. For individuals with PNES, the relationship between thyroid hormones, oxidative stress, and GABAergic modulation may influence the occurrence of these events. One primary avenue involves the disruption of the equilibrium between oxidative stress and antioxidant defenses. Hypothyroidism amplifies the production of reactive oxygen species (ROS), creating an environment conducive to oxidative stress. The brain's limited antioxidant defense system renders it susceptible to the damaging effects of ROS. Mitochondrial DNA (mtDNA), adjacent to the electron transport chain, is a major source of superoxide radicals and is especially susceptible to oxidative damage, potentially contributing to mitochondrial dysfunction, a factor implicated in seizure genesis, and, therefore, influencing the expression of PNES in susceptible individuals [6].

Another pivotal aspect involves the modulation of the GABAergic system by thyroid hormones. Severe hypothyroidism disrupts the normal function and development of GABAergic neurons, critical components of inhibitory neurotransmission. This disruption can lead to an imbalance between excitatory and inhibitory signals in the brain, increasing neuronal excitability and fostering conditions favorable for the initiation and propagation of seizures. For individuals with PNES, the altered neurological state due to heightened oxidative stress from hypothyroidism and compromised GABAergic function may trigger or exacerbate PNES episodes. Therefore, oxidative stress-induced damage and mitochondrial dysfunction could impact overall neural function, while concurrent disturbances in the GABAergic system may disrupt inhibitory control, creating conditions conducive to the development of both epileptic and psychogenic manifestations such as PNES [6].

The prevalence of spontaneous hypothyroidism during pregnancy is estimated at 2-3%, with 0.3-0.5% presenting as overt hypothyroidism and 2-2.5% as subclinical hypothyroidism. Conversely, overt hyperthyroidism affects 0.1-0.4% of pregnancies. As the second most common endocrine disorder during pregnancy, thyroid disease significantly impacts the physiological dynamics of pregnant females [5]. Post-partum thyroiditis, with a prevalence of approximately 8%, results from subclinical autoimmune thyroiditis exacerbated post-pregnancy. It most often emerges within the first year post-delivery, particularly in females without prior thyroid disease [4]. This patient's thyroid issues during pregnancy and her symptoms in the postpartum period mirror the well-documented effects of thyroid dysfunction during and after pregnancy, highlighting its significance in her case.

The development of post-partum Hashimoto's thyroiditis can occur due to a subsequent rebound from pregnancy-induced immunosuppression. During pregnancy, immune adaptations marked by increased CD4+ and CD25+ regulatory T cells lead to both T and B cell suppression [11]. Notably, approximately 20% of post-partum thyroiditis patients progress to classical Hashimoto's disease in later years, highlighting the potential development of Hashimoto's thyroiditis during and after pregnancy [12]. The development of post-partum thyroiditis in our patient, along with positive TPO antibodies, suggests a potential autoimmune basis for her thyroid and seizure-related symptoms.

## Conclusions

This case highlights a relatively rare presentation of severe post-partum hypothyroidism manifesting as PNES and myopathy and demonstrates the complex interplay between endocrine and psychogenic disorders. Her psychological history, combined with her thyroid condition, likely played a key role in triggering or exacerbating her PNES symptoms. The lack of epileptiform activity on her EEG and the quick improvement after starting thyroid hormone therapy emphasizes the importance of thoroughly evaluating endocrine etiologies in similar cases. This case serves as a reminder of the need for a wide-ranging differential diagnosis, particularly in peripartum females showing neurological and psychiatric symptoms.
